# Chondroblastic osteosarcoma of the distal tibia: a rare case report

**DOI:** 10.11604/pamj.2017.27.11.12418

**Published:** 2017-05-05

**Authors:** Aymen Ben Fredj, Lassaad Hassini, Aymen Fekih, Mohamed Allagui, Issam Aloui, Abderrazek Abid

**Affiliations:** 1 Department of Orthopaedic Surgery, University Hospital, Monastir, Tunisia

**Keywords:** Osteosarcoma, chondroblastic, tibia

## Abstract

Chondroblastic osteosarcoma, representing about 25% of osteosarcoma, is a fatal primary malignancy of the skeleton if not diagnosed and treated appropriately. It most commonly occurs in the long bones of the extremities near the metaphyseal growth plates. In this report, we describe the occurrence of chondroblastic osteosarcoma involving the left distal tibia in a 14-year-old male. The diagnosis was confirmed by the histological examination of a surgical biopsy. The patient was treated by both surgery and neoadjuvant chemotherapy. No recurrence was noted at 3 years of follow-up. To our knowledge, only two cases describing chondroblastic osteosarcoma of the distal tibia had been reported through English medical literature. Therefore, the aim of our article is to make the clinician aware of this rare clinical presentation and also to provide a comprehensive review of the literature related to this uncommon malignant tumour.

## Introduction

Chondroblastic osteosarcoma (CO) is a variant of osteosarcoma which are the most common malignant tumours of the skeleton [1, 2]. It represents about 25% of all osteosarcomas [1, 2]. It most commonly occurs in the long bones of the extremities near metaphysical growth plates [1, 2]. Although, it has been described in other bones including mandible, clavicle, cuboid and pelvis [1–5]. Rarely, CO has been reported to involve extra skeletal tissues, including breast, lung and intradural matter [1, 2, 6]. On the other hand, CO of the distal tibia had been reported only in two cases through English medical literature [2, 5]. So, herein we report the third case of CO involving the left distal tibia and we discuss the clinical features, the diagnosis and the management of this uncommon malignant tumour.

## Patient and observation

**Figure 1:**
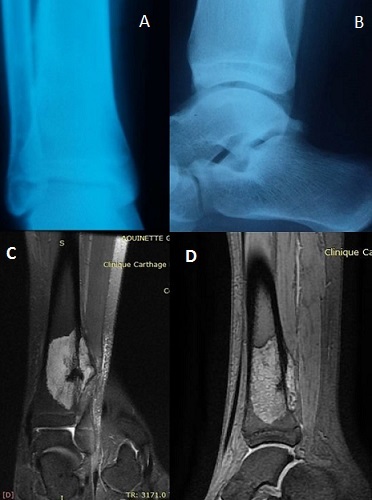
(A,B) plain radiographs showing a metaphyseal osteolytic lesion of the distal left tibia; (C,D) MRI, an extension to the adjacent soft tissue and no epiphyseal involvement

**Figure 2:**
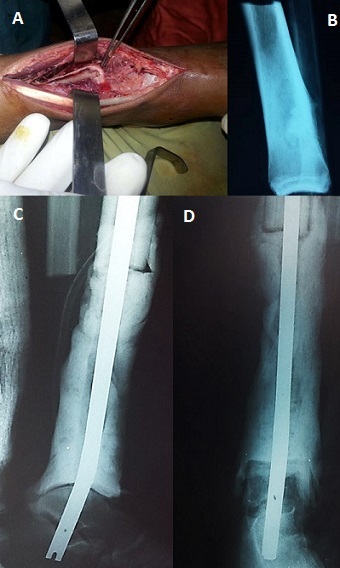
(A) per operative aspect; (B) a wide resection of tibia and fibula along 12 cm from the tibio-talar joint performed with a tumour free margin of 3 cm; (C,D) post-operative plain radiographs

**Figure 3:**
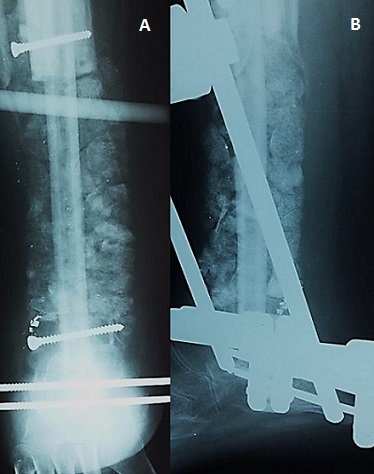
(A, B) plain radiographs post reconstruction

**Figure 4:**
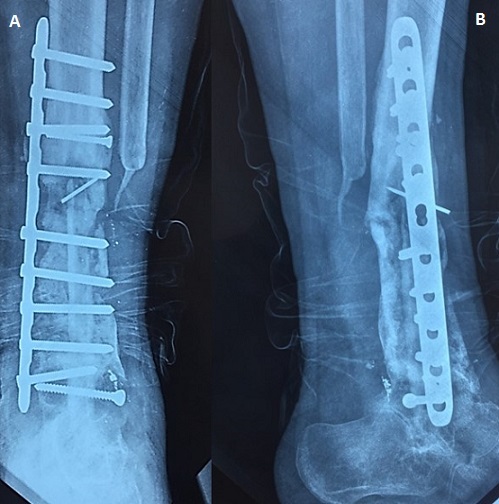
(A, B) plain radiographs after osteosynthesis with plate

A 14-year-old boy, with no medical history, consulted our out-patient department for pain in his left leg evolving 3 months ago. There was no history of trauma. He hadn't fever, nor weight loss. On examination, we noted a painful swelling in the distal end of the tibia. There was no abnormal mobility in the distal end of left tibia. The plain radiographs showed a metaphyseal osteolytic lesion of the distal left tibia (Figure 1). A Magnetic Resonance Imaging (MRI) was also demanded in order to define the extent of marrow involvement. It showed an extension to the adjacent soft tissue and no epiphyseal involvement (Figure 1). Based on his persistent pain, as well as the clinical and radiographic findings and after discussion with his parents, the patient underwent surgical biopsy. The histological examination revealed chondromyxoid matrix and hypercellular neoplasm with lobular configuration suggestive of chondroblastic type of osteosarcoma. So, the diagnosis of distal tibia CO was confirmed. A computed tomography (CT) scan of the chest was normal. The treatment was discussed in a multidisciplinary consultation meeting involving our team, the paediatrics and the oncologists. The therapeutic decision was both surgical and neoadjuvant chemotherapy including doxorubicin, cisplatin, and high-dose methotrexate. The line of resection was established with the pre-operative imaging studies. We opted for the standard anterior-lateral approach to the distal end of tibia (Figure 2). After parents' consent, we did a wide resection of tibia and fibula along 12 cm from the tibio-talar joint performed with a tumour free margin of 3 cm (Figure 2). It was send for histopathological examinations which confirmed the diagnosis of CO. The sutures were removed after 2 weeks. The post-operative chemotherapy was started after the suture removal. The skin around the surgical scar was found healthy. A reconstruction of the distal tibia was made 3 months after tumor resection (Figure 3). The patient was monitored regularly. Four months after reconstruction, we noted, through radiological control, a delay of consolidation. So, we opted for treatment by osteosynthesis with plate (Figure 4). The repeat CT scan of the chest was normal. No recurrence was noted at 3 years of follow-up.

## Discussion

We reported an uncommon location of CO. In fact, this type of malignant tumor, like other conventional osteosarcomas, has a distinct predilection for the metaphysis of long bones and most commonly affects the knee region, especially the distal femur and proximal tibia [1–5]. Located in the distal tibia, had been reported in only 2 observations [2, 5]. The first one was reported in 2009 by Jerome et al [5]. The second one was described by VandenBussche et al who had done a retrospective review of the cytopathology archives of 2 large tertiary care centers for a 15-year period (2001-2015) revealing 17 cases of CO among them only one case was located in distal tibia [2]. We have summarized the characteristics features of these two cases compared with our patient in the Table 1. On the other hand, conventional osteosarcoma accounts for approximately 90% of osteosarcomas and approximately 25% of conventional osteosarcomas are CO [1–5]. Other subtypes of conventional osteosarcoma are osteoblastic and fibroblastic osteosarcomas [7]. Nonconventional osteosarcomas include other variants: small cell, telangiectatic, periosteal, parosteal and low-grade central osteosarcomas [7]. The CO has a slight predilection for males as well as our case [3]. In the literature, the prevalence peaks are reported average age is typically within the second and third decade [2]. A relatively rate increase of CO in adolescence was described due to the growth spurt which can be related to our patient [1, 3, 5]. A genetic predisposition to osteosarcoma, was evoked by some authors but not reported in those located in distal tibia [5]. Clinically, osteosarcoma usually presents as a painful swelling which was the case of both our patient and the case reported by Jerome et al [5].

**Table 1:** The characteristics features of the two patients with CO in distal tibia compared to our case reported

Case	Age (Years)	Sex	Clinical presentation	Radiology diagnosis	Treatment	Size cm	Follow up
Jerome et al	10	Male	Painful swelling	Codman triangle Sunburst appearance	Sugery and chemotherapy	10.5	2 years: alive and doing his normal daily activities
VandenBussche et al	17	Male	Fracture	Likely osteosarcoma	Sugery and chemotherapy	13.6	4 years: death
Our case	14	Male	Painful swelling	Metaphyseal osteolytic lesion	Sugery and chemotherapy	12	3 years: alive with no recurrence

However, in the case described by VandenBussche et al, the patient suffered from pathologic fracture [2]. The radiological appearance depends on the amount of mineralization. It can show either sclerotic or lytic as well as in our case reported or mixed lesions [3, 5]. In the case reported by Jerome et al, they described the characteristic Codman triangle corresponding to the elevation of the periosteum [5]. They, also, reported the « sunburst appearance » as the result of the extension of tumour through the periosteum [5]. Usually, the tumour margins are poorly defined and the associated cortex is destroyed, which lead to soft tissues invasion [1]. In our case, the radiological aspects caused confusion with chondro-myxoid fibroma but the extension to the soft tissue revealed by MRI was in favour of malignant tumour. Moreover, some studies have pointed to the superiority of MRI and CT scans over plain film radiographs for delineation of tumour extension [**5**]. The treatment of CO usually entails total surgical resection of the tumour, combined with preoperative and postoperative chemotherapy [5]. In our patient and the two reported cases, the treatment consisted in surgery and neoadjuvant chemotherapy [2, 5]. The chemotherapeutic drugs most active in osteosarcoma are doxorubicin, cisplatin, and high-dose methotrexate [8]. In addition, many other therapies are being evaluated [5, 8]. Some authors suggested that CO had a better prognosis than do other variants of conventional osteosarcoma [3]. However, it can be associated with a prevalence of recurrence of 70% and metastasis of 15% [5, 9]. A number of published reports describe pulmonary tumour metastasis related to CO, a condition that is most often fatal [5, 9]. In fact, the patient reported by VandenBussche et al was dead 4 years after the diagnosis [2]. The cause of death was not define [2]. Although, in our case and in the observation of Jerome et al, the patients had a good evolution without recurrence or metastasis [5]. We had reported the longer follow up among all of these cases [2, 5].

## Conclusion

In summary, we report a case of CO of the distal tibia, which is a rare condition. The diagnosis was confirmed by the gold standard biopsy. The multi-centric approach, which included the neo-adjuvant chemotherapy, meticulous surgical resection of the tumour and postoperative chemotherapy, improved the prognosis and prevented the limb loss.

## Competing interests

The authors declare no competing interests.
